# Lippincott's Medical Dictionary

**Published:** 1899-06

**Authors:** 


### Reviews of Books.

**Lippincott's Medical Dictionary.** Prepared by RYLAND W. GREENE, with the collaboration of JOHN ASHHURST, Jr., M.D., GEORGE A. PIERSOL, M.D., and JOSEPH P. REMINGTON. Pp. xii., 1154. London: J. B. Lippincott Company. 1897.

There seems to be no end to the enterprise of publishers of medical works, for they publish enormous dictionaries which, we fear, can never give an adequate return for the trouble and expense incurred. The one before us is one of the most complete we have seen, and must have involved a great amount of labour in its compilation. We can strongly recommend it to all in need of a good medical dictionary. It is especially strong in the departments of chemistry and materia medica, in this respect excelling most of those with which we are familiar.

